# Synthesis of highly stable fluorescent poly(methacrylic acid-*co*-itaconic)-protected silver nanoclusters and sensitive detection of Cu^2+^†

**DOI:** 10.1039/d1ra03109k

**Published:** 2021-06-09

**Authors:** Guangyu Zhu, Hanjia Hu, Tao Yang, Junjun Ma, Sanjun Zhang, Xiaohua He

**Affiliations:** School of Chemistry and Molecular Engineering, East China Normal University Shanghai 200241 China xhhe@chem.ecnu.edu.cn; State Key Laboratory of Precision Spectroscopy, East China Normal University Shanghai 200241 China sjzhang@phy.ecnu.edu.cn

## Abstract

Stable fluorescent silver nanoclusters (AgNCs) were synthesized through one-step UV photoreduction using the multiple carboxyl copolymer poly(methacrylic acid-*co*-itaconic acid) P(MAA-*co*-IA) as a novel template. The fluorescence lifetime and the quantum yield of the obtained AgNCs were 1.84 ns and 8.9% in an aqueous solution, respectively. Owing to the multiple carboxyls of the protective P(MAA-*co*-IA) template, the obtained AgNCs have excellent advantages such as good dispersity, and high stability, which make them suitable for highly sensitive and selective detection of Cu^2+^ by fluorescence quenching. A good linear relationship exists between the degree of fluorescence quenching for silver nanoclusters and Cu^2+^ concentration ranging from 0 to 10 μM. The limit of detection (LOD) is 6.36 nM. The result implies that the as-synthesized AgNCs show great potential in the analysis field.

## Abstract 2

Silver nanoclusters were synthesized through one-step UV photoreduction using the multiple carboxyl copolymer poly(methacrylic acid-*co*-itaconic acid) as a novel template, which can be used for highly sensitive and selective detection of Cu^2+^ by fluorescence quenching.

## Graphical abstract

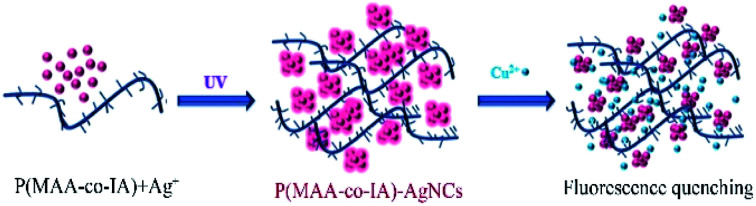

## Introduction

Fluorescent metal nanoclusters consisting of only several to tens of atoms have received great attention over the past few decades due to their huge potential in many fields, such as chemical sensing, biological catalysis, and bioimaging.^1–7^ Compared with large-size metal nanoparticles, metal nanoclusters possess an ultra-small size comparable to the Fermi wavelength of electrons, which makes them exhibit dramatically different optical, electronical and chemical properties.^8,9^ In particular, fluorescent noble metal nanoclusters have attracted special attention due to their excellent properties including good biocompatibility, low toxicity, resistance to photobleaching and a large Stokes shift.^4,10^ Ag nanoclusters (AgNCs), as one of the typical noble metal nanoclusters, are usually regarded as a representative research object due to their brighter fluorescence, higher stability, and easy synthesis.^2,7^ Generally, AgNCs can be synthesized by reducing the silver salt under the protection of templates.^11^ To date, elaborate efforts have been made to prepare water-soluble AgNCs by using different templates including DNA, thiolates and polypeptides.^4,12,13^ For example, AgNCs synthesized from DNA template stabilizer can present significantly enhanced fluorescence,^14^ and those from 2,3-dimercapto-succinic acid as a template stabilizer have an exact number of Ag atoms.^15^ Recently, Cao N. *et al.* reported the synthesis of glutathione-protected fluorescence AgNCs by the boiling water method and applied for the detection of cysteine.^16^ Various typical AgNCs which have been synthesized using different templates and applied to different fields undoubtedly have accelerated the development of nanoscience to some extent.^4,17^ Although AgNCs present stronger fluorescence than others (such as Au nanoclusters) in solution, they have some disadvantages such as weak stability and low quantum yield.^18^ To date, AgNCs synthesized by using polyelectrolyte template containing carboxylic functional groups possess high fluorescence and great stability because the carboxylic groups can not only bind the metal ions but also manipulate the conformation of the polymer according to different pH solutions.^19–22^

As we all know, copper, as one of the most fundamental trace elements and a vital cofactor for many enzymes in the human body, plays an irreplaceable role in some physiological processes.^12,13,18^ Abnormal copper content may cause negative effects on human health and even serious diseases, such as Alzheimer's disease, Parkinson's disease, and motor neuron disease.^12,23,24^ Therefore, it is more and more important to establish copper detection methods in related chemical and biological samples. Several techniques have been employed to detect Cu^2+^, such as dispersive liquid–liquid microextraction, liquid chromatography-mass spectrometry, UV-vis spectroscopy, and atomic absorption spectroscopy.^5,25–27^ The above-proposed methods could be used for effective detection in low contents. However, these methods are high-cost and complicated. Therefore, it is necessary to construct a simple and effective method for detecting Cu^2+^ with high sensitivity and good selectivity.

Herein, the stable fluorescent silver nanoclusters (AgNCs) were synthesized through one-step UV photoreduction using the multiple carboxyl copolymer poly(methacrylic acid-*co*-itaconic acid) P(MAA-*co*-IA) as a novel template. Briefly, P(MAA-*co*-IA) was first synthesized by aqueous radical polymerization, using cheap and common MAA and IA as monomers. Subsequently, the red-emitting AgNCs with high stability were synthesized through photoreduction without any reductants, which is easy to operate and eco-friendly. Finally, the as-synthesized AgNCs were further employed as a fluorescent probe to detect Cu^2+^ in the presence of other interfering metallic ions. The obtained results indicated that the AgNCs probes could be highly sensitive and suitable for Cu^2+^ detection, and the detection limit was 6.36 nM to meet the minimum requirements of the U.S. Environmental Protection Agency (EPA).^28^

## Experimental

### Materials

Methacrylic acid (MAA), itaconic acid (IA), ammonium persulfate ((NH_4_)_2_S_2_O_8_) and NaOH were purchased from Sigma-Aldrich (Shanghai, China). Silver nitrate (AgNO_3_, >99.9%) and HNO_3_ were purchased from Sinopharm Chemical Reagent Co., Ltd (Shanghai, China). Copper(ii) nitrate hydrate (Cu(NO_3_)_2_·3H_2_O) and other metallic salts were purchased from Aladdin Reagent Co., Ltd (Shanghai, China). All above chemical reagents used in the research were analytical grade and without further purification. The ultrapure water was produced by the Ultrapure Millipore water system and throughout all the experiment.

### Characterization

The UV-vis absorption spectra of the as-synthesized AgNCs aqueous solution were carried out by using a JASCO V-570 spectrophotometer at room temperature. Fluorescence measurements were operated by using a FLS 1000 fluorescence spectrophotometer (Edinburgh, England). The emission spectra of the solution were recorded upon excitation at 510 nm. Transmission electron microscopic (TEM) images were obtained *via* a JOEL JEM-2100F transmission election microscope upon an accelerating voltage of 200 kV. X-ray photoelectron spectroscopy (XPS) was achieved by means of an AXIS SURA (Kratos, UK).

### Synthesis of P(MAA-*co*-IA)

P(MAA-*co*-IA) were synthesized by radical polymerization in aqueous solution (see ESI, Scheme S1†). Typically, 3.91 g (0.03 mol) of IA and 30 mL of H_2_O were first added to a three-neck flask with a condenser and the solution was continuously stirred for 30 minutes to fully dissolve the IA. Subsequently, 5.9 mL (0.07 mol) of MAA and 0.02 g of (NH4)_2_S_2_O_8_ (dissolved in 1 mL H_2_O) were added slowly the above solution, then the solution was continuously stirred at 70 °C for 2.5 h under high purity nitrogen. The synthetic crude product was obtained by precipitating into excess cold acetone and purified by dialyzing against water for 3 days to remove the unreacted MAA, IA and (NH_4_)_2_S_2_O_8_. Finally, the P(MAA-*co*-IA) was obtained as the white solid (yield: 4.5 g). The amount of carboxylic groups on the chain segment of the P(MAA-*co*-IA) was determined according to the previous literature.^20^

### Synthesis of P(MAA-*co*-IA) protected AgNCs

P(MAA-*co*-IA)-protected AgNCs were synthesized *via* UV irradiation at 365 nm according to the literature.^29^ Briefly, P(MAA-*co*-IA) (5 mL, 0.12 M) aqueous solution and AgNO_3_ (5 mL, 0.03 M) solution were mixed and stirred in the dark for 5 min to obtain the Ag^+^–carboxylate complex. After adjusting pH value by using HNO_3_ (0.1 M) and NaOH (0.1 M), the above solution was exposed to obtain P(MAA-*co*-IA)-protected AgNCs through using a UV-irradiation within a set time. The molar ratio of the carboxyl group/Ag^+^ and pH value of the Ag^+^–carboxylate complex in a series of experiments were adjusted to evaluate to explore the optimum conditions for the synthesis of Ag nanoclusters.

### Fluorescence detection of Cu^2+^ ions

The Cu^2+^ aqueous solution with a stock concentration of 30.0 mM was prepared from copper(ii) nitrate hydrate. For the research, the Cu^2+^ aqueous solution with different concentrations were mixed into the AgNCs solution, and then incubated at room temperature for 5 minutes before the fluorescence measurements were carried out.

### Selectivity detection

Different metal ions including Ca^2+^, Cd^2+^, Fe^3+^, Mg^2+^, Zn^2+^, Pb^2+^, Co^2+^, Na^+^, Mn^2+^, K^+^ and Ni^2+^ were employed to explore the selectivity of AgNCs on them. The as-synthesized AgNCs solution mixed with the metal ions solution with appropriate concentration were incubated for 5 minutes. The fluorescence measurements were performed at the same condition.

## Results and discussion

### Synthesis of P(MAA-*co*-IA)-protected AgNCs

**Scheme 1:**
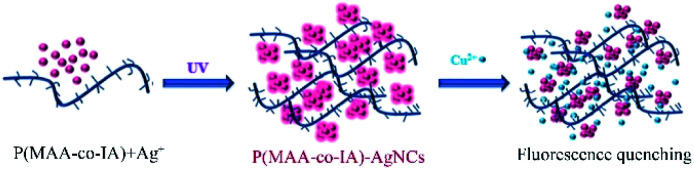
The schematic illustration of the synthesis of P(MAA-*co*-IA)-protected AgNCs and the detection of Cu^2+^.

**Fig. 1:**
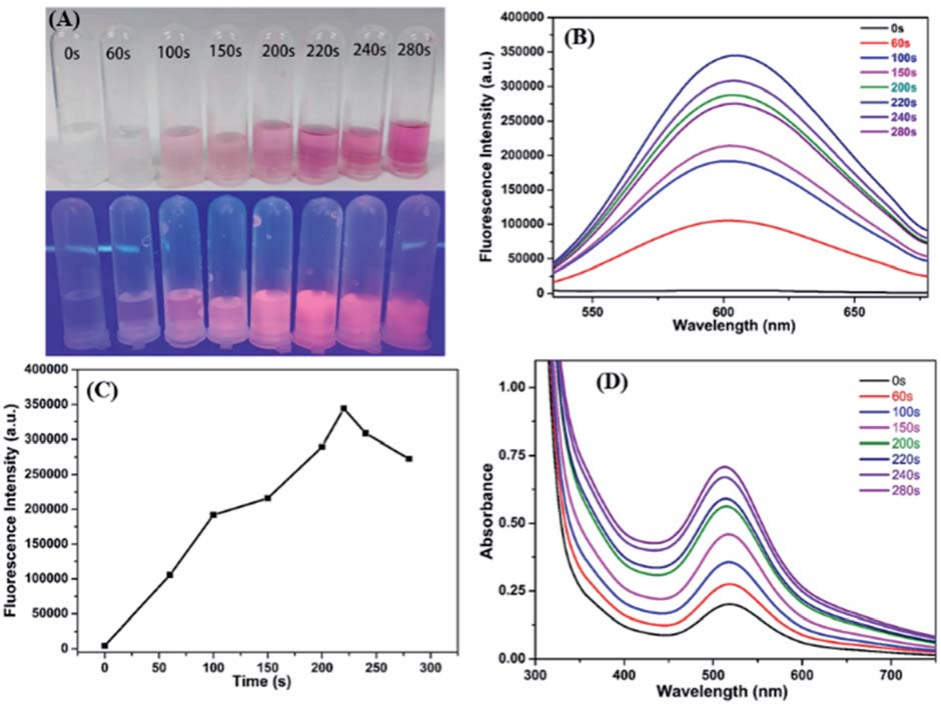
(A) The corresponding photographs of AgNCs under Vis-light (up) and UV lamp (down). (B) The corresponding emission intensity of AgNCs solution with different radiation time. (C) The corresponding emission intensity of AgNCs solution with different radiation time. (D) UV-vis absorption spectra of AgNCs.

P(MAA-*co*-IA)-protected Ag nanoclusters were synthesized by the photoreduction procedure (Scheme 1). To obtain an appropriate UV irradiation time during the synthesis of P(MAA-*co*-IA)-protected AgNCs using the photoreduction, the Ag^+^–carboxylate complex solution was irradiated by UV light (*λ* = 365 nm, power = 6 W) with different time while the molar ratio of the carboxyl group/Ag^+^ and the pH value of the Ag^+^–carboxylate complex were set as 3/1 and 5.5, respectively. Prior to UV irradiation, the mixture solution is colourless and transparent (Fig. 1A) and the fluorescence emission is hardly observed (Fig. 1B). With UV irradiation time, the colour of the mixture solution gradually changes from colourless to pink shown in Fig. 1A. The fluorescence emission intensity (the excitation wavelength = 501 nm) increases with increasing the UV irradiation time from 0 to 220 s (Fig. 1B and C), indicating the formation and the growth of AgNCs.^20,21^ When the radiation time further increases to 280 s, the fluorescence emission intensity decreases gradually, which suggests the formation of the non-luminescent silver nanoparticles.^16,30^ Therefore, 220 s is set as an appropriate UV irradiation time in the present research system. The UV-vis spectra are shown in Fig. 1D. No absorption peaks are observed in the region of 400–450 nm, indicating the formation of AgNCs rather than Ag nanoparticles.^30,31^ On the other hand, the maximum emission wavelength shift hardly with the irradiation time (Fig. 1B), which indicates that the as-synthesized AgNCs are relatively uniform.^20^

**Fig. 2:**
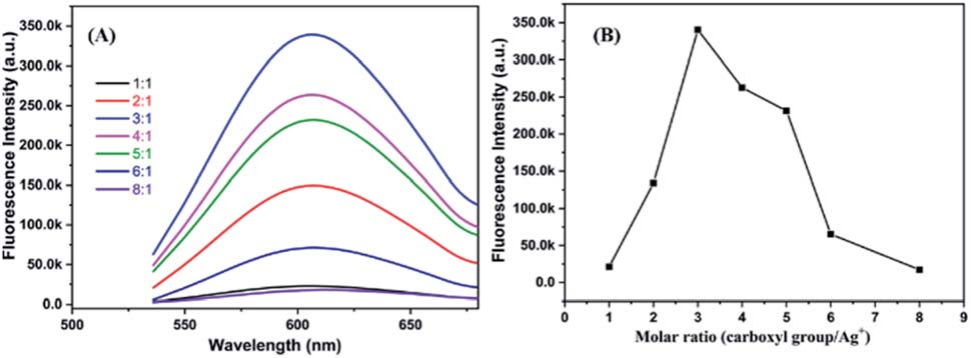
Fluorescence emission spectra (A) and fluorescence intensity value (B) of AgNCs solution synthesized by using different molar ratio of COO^−^/Ag^+^.

**Fig. 3:**
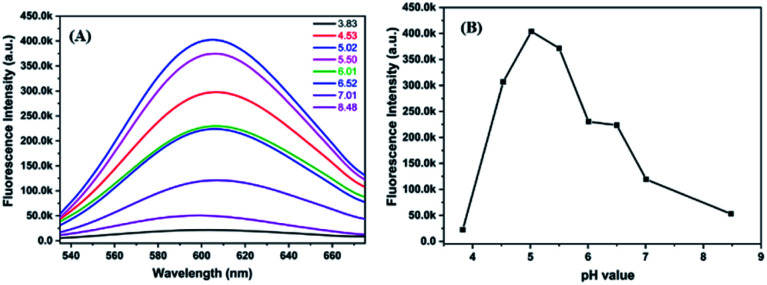
Fluorescence emission spectra (A) and fluorescence intensity value (B) of AgNCs solution synthesized at different pH values.

The molar ratio of precursors plays a crucial role in the formation of the metal nanoclusters.^4,17^Fig. 2A shows the fluorescence emission spectra of P(MAA-*co*-IA)-protected AgNCs using the different molar ratio of the carboxyl group/Ag^+^ under UV irradiation time of 220 s and the pH values of 5.5. The fluorescence emission intensity increases with increasing the molar ratio of the carboxyl group/Ag^+^ from 1/1 to 3/1, and then decreases gradually with increasing that from 3/1 to 8/1 (Fig. 2). The maximum fluorescence intensity can be observed at the molar ratio of the carboxyl group/Ag^+^ of 3/1 in our research system. The photoluminescence mechanism of AgNCs is based on the transfer of electron from the oxygen atom of the ligand to Ag^+^ and then to the silver atoms.^29^ When the molar ratio of the carboxyl group/Ag^+^ is too low, the transfer of the electron from ligand to metal irons is restrained and weaken the fluorescence emission intensity of AgNCs. On the other hand, if the molar ratio of the carboxyl group/Ag^+^ is too high, the formation of AgNCs can be prohibited. Therefore, the optimal molar ratio of the carboxyl group/Ag^+^ is 3/1 in our research system. Like any templates containing carboxylic acid, P(MAA-*co*-IA) possesses also typical pH-responsive.^11,20^ Therefore, the formation of the AgNCs protected by P(MAA-*co*-IA) was easily regulated by the pH values of the solution. Fig. 3A shows the fluorescence spectra of AgNCs synthesized at the same irradiation time (220 s) and the carboxyl group/Ag^+^ molar ratio of 3/1 but different pH values from 3.83 to 8.48. It is easy to observe that the emission intensity increases with the pH values varying from 3.83 to 5.02, and then decreases with increasing those from 5.02 to 8.48 (Fig. 3B). The strongest fluorescence intensity is obtained when the pH value of the solution is 5.02 in our research system. When the pH value is below 3.83, the fluorescence emission is almost invisible, meaning that no fluorescent AgNCs are formed. When the pH value of the mixture solution is more than 5.50, the fluorescence emission intensity of the synthesized AgNCs decreases rapidly (Fig. 3B). The pH value of the solution mainly affects the deprotonation of the carboxylic acid groups, thus affecting the binding between the carboxylic acid group and Ag^+^. At the low pH values (<3.83), the slight deprotonation of carboxylic acid groups (p*K*_a_ = 4.25), meaning the low COO^−^ concentration, do not favour the formation of fluorescent Ag nanoclusters.^21^ When the pH is in the range of 5.02–5.50, the carboxylic acid groups are almost completely deprotonated,^21,32^ which favour the binding between carboxylic acid groups and Ag^+^. As a result, the formation of more AgNCs is beneficial to enhance the fluorescence emission intensity. In the basic solution (>6.0), the color of the reaction mixture turns from colorless to brown, indicating the formation of AgOH.^20^ Thus, the optimal conditions for the synthesis of fluorescent AgNCs were as follows: the irradiation time is 220 s, the molar ratio of precursor is 3/1, and the pH value of the solution is 5.02.

**Fig. 4:**
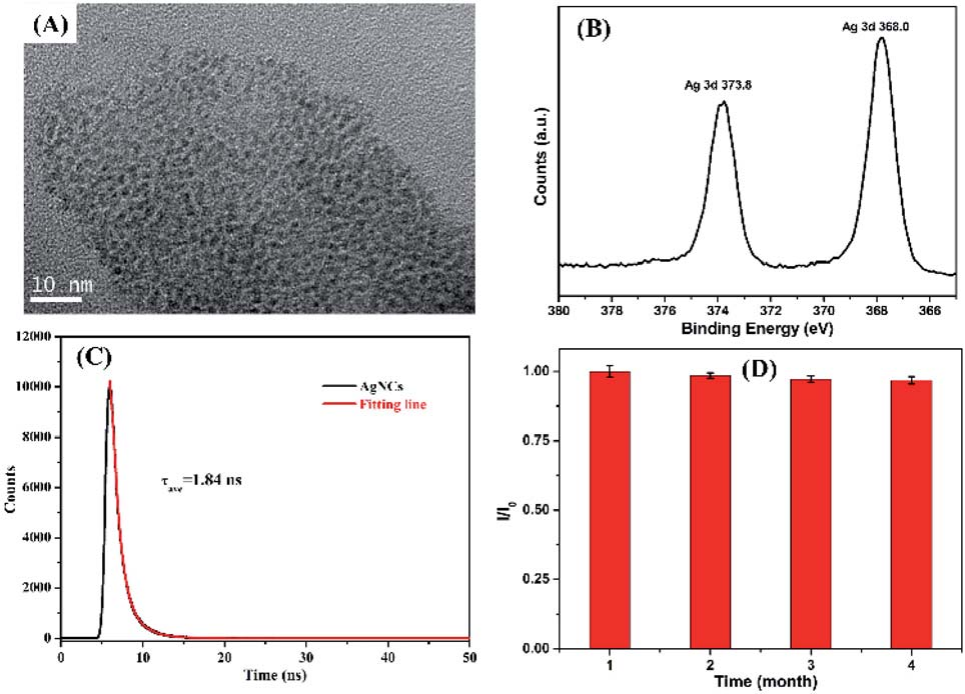
P(MAA-*co*-IA)-protected AgNCs: (A) TEM image, (B) XPS spectrum of the Ag 3d region, (C) fluorescence decay trace of AgNCs and the curve fitting line (red line), (D) the storage time (*I*_0_ and *I* represent the fluorescence intensity of AgNCs before and after the storage time, respectively).

Fig. 4A shows the TEM image of P(MAA-*co*-IA)-protected AgNCs obtained under the optimal experiment conditions. The uniform Ag particles with a diameter of approximately 2.1 nm (see ESI, Fig. S1A†) are formed. At the same time, XPS is employed to further confirm the formation of the as-synthesized AgNCs, as shown in Fig. 4B. The typical energy values of metallic Ag can be obviously observed, indicating that Ag^+^ ions are successfully reduced to metallic Ag (0) at the optimal conditions.^30^ The as-synthesize AgNCs exhibit excellent fluorescent property (see ESI, Fig. S1B†), their fluorescence lifetime and quantum yield are 1.84 ns and 8.9% (Fig. 4C), respectively. On the other hand, the fluorescence intensity of the as-synthesized AgNCs in the aqueous solution are almost unchangeable (Fig. 4D) after stored at 4 °C in a refrigerator, which indicates the excellent stability of AgNCs. The reasons can be attributed to the template ligand, P(MAA-*co*-IA), containing more carboxyl groups of per repeat unit in the backbones and providing more chelate sites for Ag^+^ ions, which is beneficial to the formation and stabilization of Ag nanoclusters. The obtained results confirm that the silver nanoclusters have been successfully synthesized through using P(MAA-*co*-IA) as the template.

### Cu^2+^ detection using the AgNCs as fluorescent probes

**Fig. 5:**
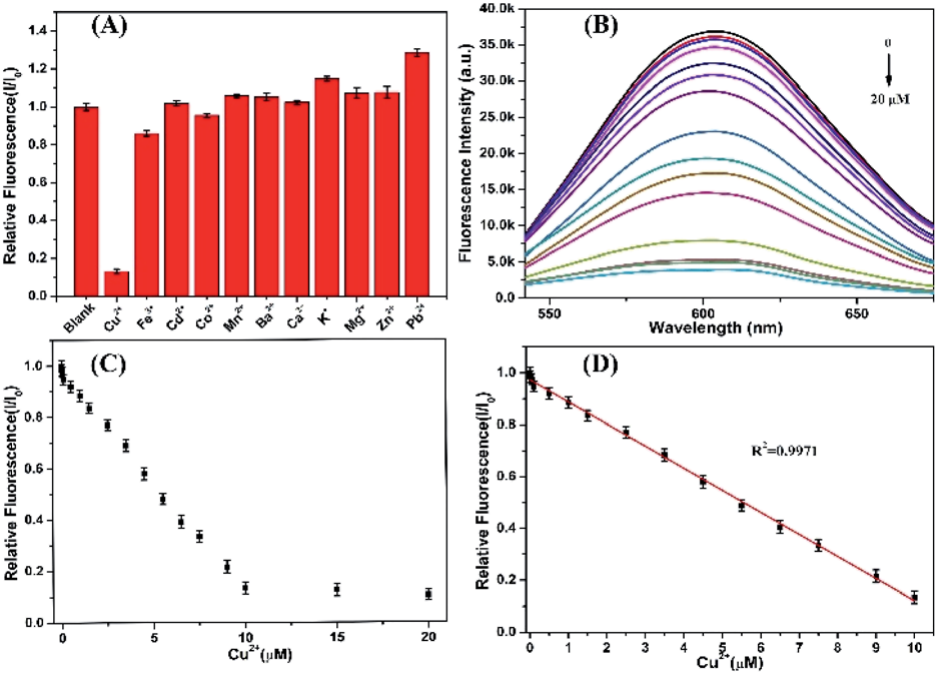
(A) The fluorescence changes of AgNCs in the different metal ions. The concentrations of all metallic ions are 10 μM. *I*_0_ and *I* represent the fluorescence intensity of AgNCs in the absence and in the presence of metallic ions. (B) Fluorescence emission spectra of AgNCs incubated with different concentrations of Cu^2+^. (C) Plots of *I*/*I*_0_*versus* the concentrations of Cu^2+^ varying from 0 to 20 μM. (D) Linear correlation curve of AgNCs incubated with different concentrations of Cu^2+^ ranging from 0 to 10 μM.

The as-synthesized AgNCs show highly strong fluorescent emission which can be served as probes to detect the heavy metallic ions. Studies were first performed to investigate the effects of different metallic ions, including Ba^2+^, Ca^2+^, Cd^2+^, Co^2+^, Cu^2+^, Fe^3+^, K^+^, Mg^2+^, Zn^2+^, Mn^2+^, and Pb^2+^, on the fluorescence intensity of P(MAA-*co*-IA)-protected AgNCs solution.^19,33^ As shown in Fig. 5A, the fluorescence intensity of the AgNCs is greatly quenched in the presence of Cu^2+^, while that of the AgNCs is almost unchangeable in the absence of Cu^2+^(see ESI, Fig. S2†). Therefore, the AgNCs can be used as probes for the detection of Cu^2+^. To further evaluate the sensitivity of the fluorescent probes AgNCs, the fluorescent emission intensity of the AgNCs solutions with different concentrations of Cu^2+^ were monitored. Fig. 5B shows the fluorescent intensity change of the AgNCs at 607 nm with increasing the titration of Cu^2+^ from 0 to 20 μM. The fluorescent intensity of the AgNCs decreases gradually with the increase of Cu^2+^ concentration and a good linear correlation (*R*^2^ = 0.9971) exists between the quenching degree of fluorescence and the concentrations of Cu^2+^ ranging from 0 to 10 μM as shown in Fig. 5C and D. The limit of detection (LOD) of Cu^2+^ is evaluated to be 6.36 nM (S/N = 3), which is greatly lower than the allowable maximum levels of Cu^2+^ in drinking water permitted by the EPA (∼20 μM).^34^ The results confirm that the obtained fluorescent AgNCs probes have great potential for the sensitive detection of copper ions. The mechanism of fluorescence quenching between AgNCs and Cu^2+^ can be attributed to the binding between Cu^2+^ and the free carboxyl group, and attaching to the surface of the fluorescent silver nanoclusters, which hinders the electron transfer in the system and leads to fluorescence quenching (Scheme 1).

In addition, further studies were performed to explore the resistance of the as-synthesized AgNCs to the interference from other metallic cations. The fluorescence of the as-synthesized AgNCs solution was first quenched by Cu^2+^ (10 μM), and then different metallic cations (50 μM) including Ba^2+^, Ca^2+^, Cd^2+^, Co^2+^, Fe^3+^, K^+^, Mg^2+^, Zn^2+^, Mn^2+^, and Pb^2+^ were again added. The ratio of the relative fluorescence (*I*/*I*_0_) of the as-synthesized AgNCs solution with Cu^2+^ co-existing before (*I*_0_) and after (*I*) other metallic ions were almost unchangeable expect for Co^2+^ and Fe^3+^causing approximately 10% decrease due to the possible aggregation of silver nanoclusters, and except for Ba^2+^, Ca^2+^ and Pb^2+^ causing slightly increase due to their stronger interactions with silver nanoclusters.^28^ The obtained results proves that the as-synthesized fluorescent AgNCs have sensitivity and selectivity for the detection of Cu^2+^ and other common metallic cations have little effects on it.

### Real sample analysis

To investigate the feasibility for detecting Cu^2+^ in real samples, the as-synthesized AgNCs was applied for the detection of the Cu^2+^ content in a tap-water sample filtered by a filter of 0.22 μM. According to the standard calibration curve method (see ESI, Fig. S4†), the concentration of Cu^2+^ in a real water sample can be detected and calculated to be 6.36 nM. The recovery percentage is about 100.0% (see ESI, Table S1†). Compared with the previous report on the detection of Cu^2+^ through this method using Ag nanoclusters as a fluorescent probe (see ESI, Table S2†), the as-synthesized AgNCs exhibits a good feasibility and sensitivity for the Cu^2+^ detection in the tap-water sample.

## Conclusions

In summary, novel water-soluble Ag nanoclusters were successfully synthesized through one-step UV photo-reduction using the multiple carboxyl copolymer P(MAA-*co*-IA) as the template. The as-synthesized AgNCs possess good fluorescence and high stability. At the same time, they have great sensitivity and selectivity for Cu^2+^ which is further applied to the detection of Cu^2+^ in a tap-water sample. The findings suggest that the as-synthesized AgNCs show great potential in the analysis field.

## Author contributions

Guangyu Zhu and Hanjia Hu contributed equally to this work. All authors have given approval to the final version of the manuscript.

## Conflicts of interest

There are no conflicts to declare.

## Supplementary Material

RA-011-D1RA03109K-s001
