# Supplementary material for: Synthesis of highly stable fluorescent poly(methacrylic acid-co-itaconic)-protected silver nanoclusters and sensitive detection of Cu2+
Source: RSC Adv. 2021 Jun 9;11(34):20720–4. doi: 10.1039/d1ra03109k (PMC9034000; doi:10.1039/d1ra03109k)
Supplement: RA-011-D1RA03109K-s001 [file RA-011-D1RA03109K-s001.pdf]

Electronic Supplementary Material (ESI) for RSC Advances.
This journal is © The Royal Society of Chemistry 2021

Electronic Supplementary Information (ESI)

Synthesis of highly stable fluorescent poly(methacrylic acid-co-itaconic)-protected silver nanoclusters and sensitive detection for Cu^{2+}

Guangyu Zhu,[a] Hanjia Hu,[b] Tao Yang,[a] Junjun Ma,[a] Sanjun Zhang,[b]* Xiaohua He[a]*

[a] School of Chemistry and Molecular Engineering, East China Normal University, Shanghai 200241.

[b] State Key Laboratory of Precision Spectroscopy, East China Normal University, Shanghai 200241.

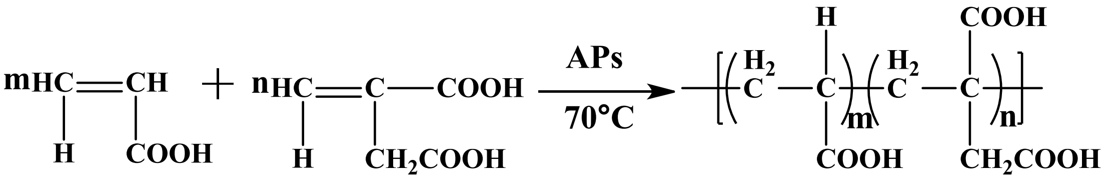

Scheme 1S The synthesis route of P(MAA-co-IA)

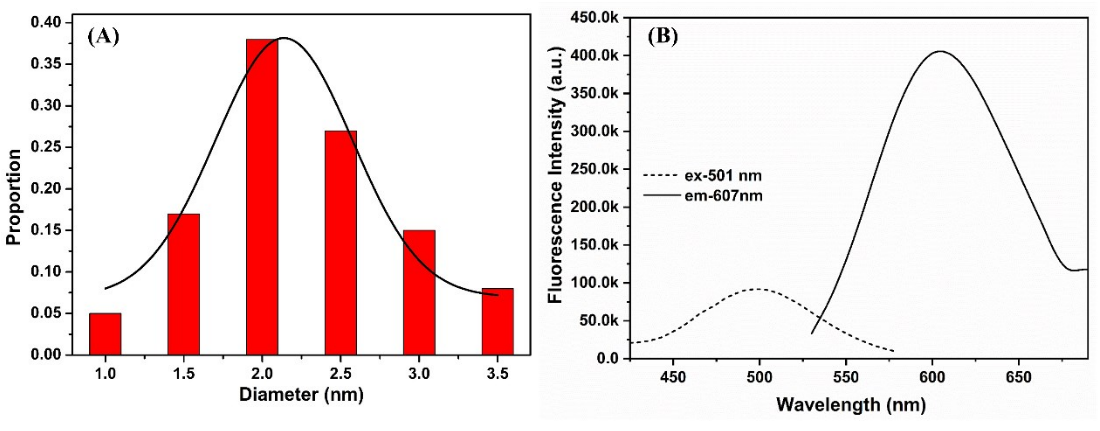

Fig. S1 P(MAA-co-IA)-protected AgNCs: (A) The size profile from SEM. (B) Fluorescence emission and excitation spectra (λ_{ex}=501 nm). The synthesis conditions: the irradiation time 220 s; the molar ratio of precursor, 3/1; the pH value of the solution, 5.02.

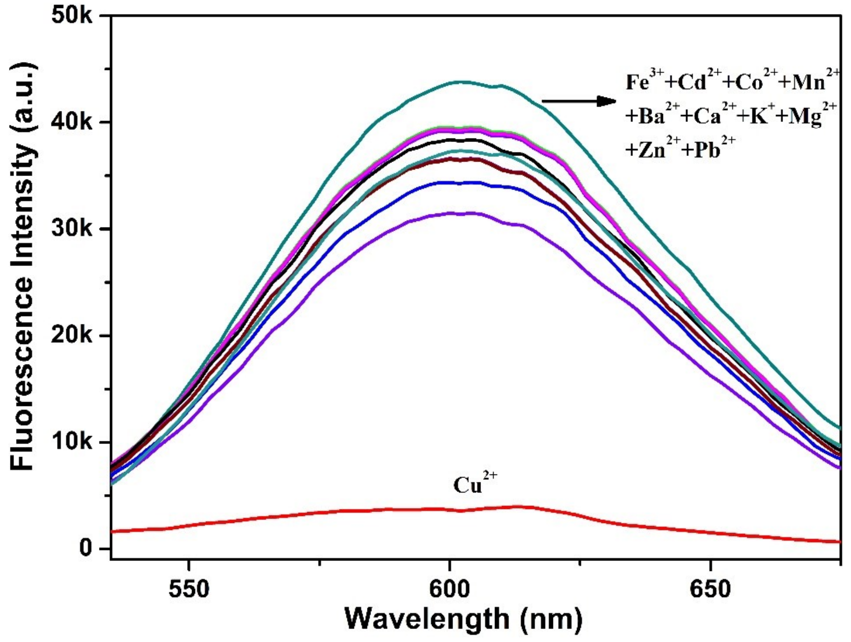

Fig. S2 Fluorescence spectra of P(MAA-co-IA)-protected AgNCs in the presence of different mental ions in aqueous solution.

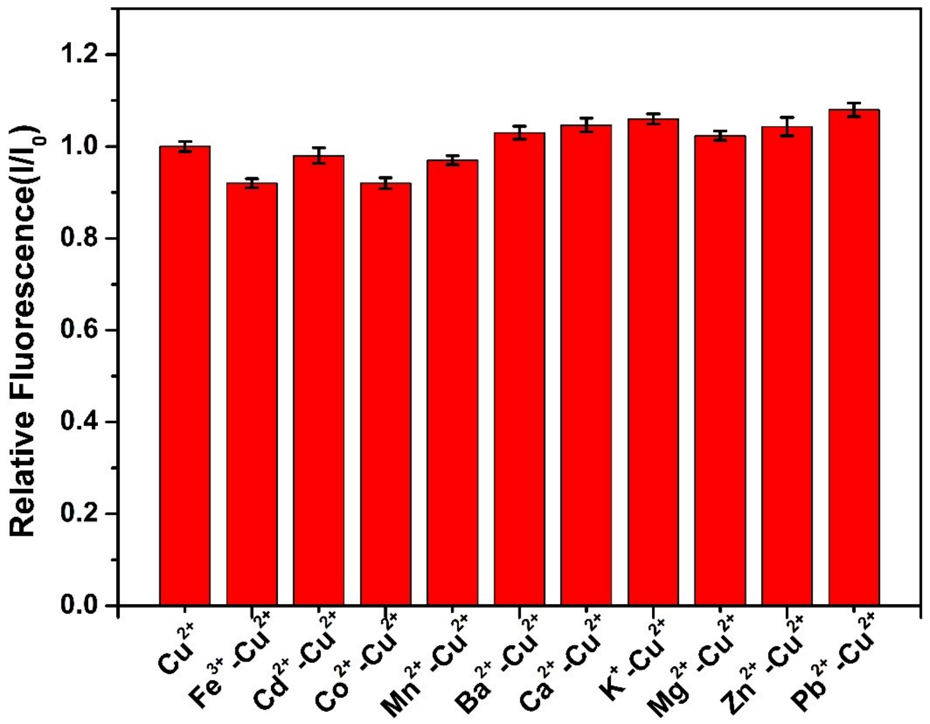

Fig. S3 Fluorescence changes of Ag NCs quenched by Cu^{2+} in the presence of different mental ions. The concentrations of other metallic ions except for Cu^{2+} (10 μM) are 50 μM. I_0 and I represent the fluorescence intensity of AgNCs in the absence and in the presence of other metallic ions.

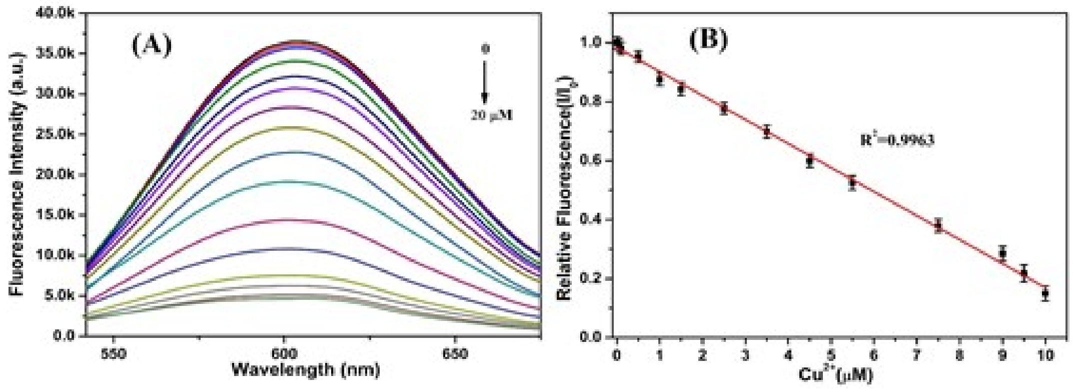

Fig. S4 (A) The fluorescence emission changes of AgNCs incubated with different concentrations of Cu^{2+} prepared through the ultrapure water. (B) The standard calibration curve based on the relative fluorescence intensity of AgNCs versus the concentration of Cu^{2+}. I_0 and I respectively represent the fluorescence intensity of AgNCs before and after the addition of Cu^{2+} aqueous

solution.

Table S1 Determination of Cu^{2+} in tap-water samples.

Sample	Added (μmol/L)	Found (μmol/L)	Recovery (%)	RSD (%, n=3)
Tap water 1	1.0	0.97	97.00	3.21
Tap water 2	3.0	3.01	100.33	1.36
Tap water 3	5.0	5.06	101.20	1.57
Tap water 4	7.0	6.99	99.86	2.31

Table S2 Detection performance of Cu^{2+} based on analysis method of different fluorescent nanomaterials

Nanomaterials	Linear range	Detection limit	Reference
PEI-Ag NCs	10nM -7.7 μL	10 nM	1
H_2L	110nM -3 μL	474 nM	2
DNA-Cu/Ag NCs	10 nM – 5 μL	5 nM	3
DHLA-Ag NCs	78 nM - 1500nM	34 nM	4
Lys-Au NCs	10 nM -7 μL	3 nM	5
DNA-Ag NCs	10 nM - 200 nM	8 nM	6
P(MAA-co-IA)-Ag NCs	0 - 10 μL	6.36 nM	This paper

References

1. Z. Yuan, N. Cai, Y. Du, Y. He and E. S. Yeung, *Anal. Chem.*, 2014, **86**, 419-426.
2. G. I. Mohammed, H. A. El-Ghamry and A. L. Saber, *Spectrochim. Acta A Mol. Biomol. Spectrosc.*, 2021, **247**, 119103.
3. X.-F. Huang, B.-X. Ren, C.-F. Peng, X.-L. Wei and Z.-J. Xie, *Microchem. J.*, 2020, **158**, 105214.
4. S. H. Ren, S. G. Liu, Y. Ling, N. B. Li and H. Q. Luo, *Spectrochim. Acta A Mol. Biomol. Spectrosc.*, 2018, **201**, 112-118.
5. Y. Xu, X. Yang, S. Zhu and Y. Dou, *Colloids and Surfaces A: Physicochem. Eng. Aspects*, 2014, **450**, 115-120.

6. G. Y. Lan, C. C. Huang and H. T. Chang, *Chem. Commun.*, 2010, **46**, 1257-1259.
